# Epidemiology and Direct Medical Cost of Osteoporotic Hip Fracture in Chile

**DOI:** 10.1155/2020/5360467

**Published:** 2020-03-25

**Authors:** Iván Quevedo, Juan C. Ormeño, Bunio Weissglas, Cristóbal Opazo

**Affiliations:** ^1^Endocrinology Section, Department of Internal Medicine, School of Medicine, University of Concepción, Concepción, Chile; ^2^School of Medicine, University of Concepción, Concepción, Chile

## Abstract

The osteoporotic hip fracture is associated with a high impact on morbidity, mortality, and health expenditure. The Chilean health system is made up of a mixed care system, with the public system called FONASA and the private system called ISAPRE. The people with lower incomes are listed on FONASA and correspond to 80.8% of the population. The aims of this study were to describe the incidence of hip fracture in the Chilean population from the age of 45 years and to estimate the direct medical cost of this disease. The records of the Department of the Health Statistics and Information of the Ministry of Health were used, from which the number of national hospital discharges due to hip fractures was obtained (codes S720, S721, and S722 of the ICD-10), in adults aged 45 years or older, by sex, from 2006 to 2017. The cost of osteoporotic hip fracture treatment in the public health system was obtained from the data of the surgical treatment according to the payment method associated with diagnosis (PAD bonus). A surgical intervention budget was used in a private clinic to calculate the direct cost of osteoporotic hip fracture in the private system. Between 2006 and 2017, the number of hospital discharges due to osteoporotic hip fracture in adults aged 45 years and older has increased progressively, registering 9.583 hospital discharges for this cause in 2017, which corresponds to 50% more than those recorded in 2006, with a 3 : 1 F/M ratio. The mean annual rate of hip fractures is 148.7 per 100,000 inhabitants aged above 45 years. The individual cost of managing an osteoporotic hip fracture in the public system was USD$ 3,919, and USD$ 9,092 in the private health system. The incidence of hip fracture was comparable with data from Southern European countries and from neighboring countries, such as Argentina and Uruguay. Hospitalization cost of hip fracture in Chile was 34 million USD per year. Hip fracture constitutes a serious healthcare problem in Chile, and efforts for the prevention and management of osteoporosis are needed.

## 1. Introduction

The Chilean population is in an accelerated period of aging. In 2017, the Chilean population had 17.5 million inhabitants, of which 16.2% (2.8 million) were 60 years old or older [1]. The aging index, which corresponds to the people aged 65 years and older in proportion to the people under 15 years old, shows that by 2050, the proportion is projected to be 177 persons of 65 years old and older per 100 persons under 15 years old [2]. It is estimated that by 2050, the total population of the country will be 21.6 million inhabitants, of which 32% (6.9 million) will be 60 years old or older [2].

One of the most prevalent pathologies in older adults, potentially causing them greater disabilities, is osteoporosis, a systemic skeletal disease characterised by a reduction in bone mass, with the consequent increased risk of fracture [3]. Women have the highest prevalence of osteoporosis worldwide, as a result of the decrease in estrogen levels after menopause, with an estimated 200 million women affected worldwide [4, 5].

Vertebral, wrist, and hip fractures are considered the classic osteoporotic fractures [6], being the hip fracture the one with the greatest economic impact. Although it represents only 18% of all osteoporotic fractures, it represents more than 40% of the health expenditure associated with osteoporotic fractures [5, 7]. In 2000, approximately 9 million osteoporotic fractures occurred worldwide, and of these over 70% affected were women [5, 7].

The economic cost of hip fracture is a relevant issue that has been studied in developed countries [5], given that the cost of fragility fractures is estimated to be higher than that of other diseases with high disabling potential such as Parkinson's disease, rheumatoid arthritis, and stroke [8]. In relation to hospitalizations, the same phenomenon is seen: since a Swiss study reported that the incidence of hospitalizations by osteoporosis and its complications is twice as much as breast cancer, acute myocardial infraction, and stroke hospitalizations, three times higher than those associated with chronic obstructive pulmonary disease, and 6 times higher than those associated with diabetes [9].

A systematic review of 113 studies from 1993 to 2015, which included more than 670,000 patients, reported that the approximate cost of the first year after suffering a hip fracture is $43,000 dollars, with the approximate cost of hospitalization being $10,000 dollars [10]. In 2010, the cost associated with osteoporosis in Europe was estimated to be 37 billion euros, representing 66% of the cost associated with fracture management and 29% of the cost of postfracture care [5, 11]. At 2025, the total costs associated with osteoporosis in North America are expected to be 25 billion dollars, with an estimated 3 million osteoporotic fractures per year by that date [12, 13]. In the case of Asian countries, there were 2.3 million osteoporotic fractures in China in 2010, with a total cost of 9.45 billion dollars; by 2050, 6 million fractures are estimated with a total cost of 25 billion dollars [14].

In Latin America, studies on the economic impact of osteoporotic fractures (mainly hip fracture) estimate that the annual national expenditure for this cause is 190 million dollars in Argentina [15], 256 million dollars in Mexico [16], and 97 million dollars in Brazil [17].

In Chile, in 2001, 4.937 hospital discharges were recorded due to hip fracture in adults aged 65 years or more (code S72 in the ICD-10) [18], while in 2017, 8.322 hospital discharges were recorded nationwide for the same reason in adults aged 65 years or more [19]. The Chilean Health system is made up of a mixed care system, with the public system called FONASA and the private system called ISAPRE. Generally, people with lower incomes are listed on FONASA and correspond to 80.8% of the population, and on the other hand, the 14.4% that correspond to the highest income group are listed on ISAPRE. There is 2.8% of the population with a system that is listed in the armed forces health system. The Chilean Society of Osteology and Mineral Metabolism estimated in the last decade that the direct hospital cost of treating a hip fracture ranged between 2000 and 7000 dollars depending on the type of health system to which the patient belonged (public or private) [19].

In 1991, the incidence of hip fractures in Chile was analyzed through the use of medical records. A general incidence of 23.5 fractures per 100,000 inhabitants was reported. In women aged 50 years or more, the incidence was 192.5 per 100,000 women and increased to 617 per 100,000 women in women aged 75 years or more, which is double the rate found in men [19].

The objectives of this study were to update the epidemiology of the osteoporotic hip fracture in Chile and to determine the direct economic cost of this pathology in health systems.

## 2. Materials and Methods

### 2.1. Epidemiological, Descriptive, and Retrospective Study

The records of the Department of Health Statistics and Information (DEIS) of the Ministry of Health were used, from which the number of national hospital discharges due to hip fractures was obtained, which were associated with the following causes: femoral neck fracture (code S720 of the ICD-10), pertrochanteric fracture (code S721 of the ICD-10), and subtrochanteric fracture (code S722 of the ICD-10), in adults aged 45 or more, by sex, from 2006 to 2017 [18]. The approach used here was to characterize hip fracture as osteoporotic when they are associated with low bone mass and their incidence rises with age after the age of 50 years since among the population aged over 50 years, one in three women and one in five men will suffer a fragility fracture [20, 21]. Any bone fragility hip fracture at the age of ≥50 years was considered as osteoporosis hip fracture. However, due to the classification of the population in the records of the DEIS, in which they are classified in 45–64 years old, 65–79 years old, and 80 years or older, we had to include the population aged between 45 and 50 years old because if we did not include them, we could have not included the population aged between 50 and 60 years old. The population aged between 45 and 50 years old may not have suffered fractures due to fragility, but they had to be included, or the population aged between 50 and 64 years old could not have been included.

From the estimates and projections of the population of Chile in 1992–2050, of the National Institute of Statistics (INE) [22], the data of the national population aged 45 years or more were obtained accorded to sex, between 2006 and 2017. We used these data and the hospital discharges due to hip fractures in adults aged 45 years or more between 2006 and 2017 to calculate the incidence rate of hip fracture per 100,000 inhabitants aged 45 years or more, between 2006 and 2017.

The cost of osteoporotic hip fracture treatment in the public health system was obtained from the data of the surgical treatment of the hip fracture according to the payment method associated with diagnosis (PAD bonus), code 2501040 of FONASA, which is a form of payment that allows to know in advance the total value of the account in those surgical interventions in agreement with FONASA [23]. The PAD bonus covers the entire hip fracture care, even covering additional costs of hospitalization complications.

The PAD bonus includes the following:

- Surgical treatment of the fracture of the femur (thigh), by osteosynthesis, by any technique.
- The fees of all the professional team that is technically required, under the administrative and legal responsibility of the provider in agreement.
- The values of the day beds and the right of the surgical pavilion, including the differences in rates for these concepts.
- The medications and supplies used during hospitalization.
- All the necessary services to fully resolve the pathology.
- Comprehensive care up to 15 days after the patient's discharge, including postoperative controls; the repair of involuntary lesions and the treatment of the most frequent complications derived from the resolution of the pathology.
- In case of complications, it includes the required diagnosis, treatment, and hospitalization.
- Without payment of differences for any reason to the beneficiary, or for day beds, flag right, surgical technologists, medications, or supplies.

Osteosynthesis of the hip consists in the surgical treatment of thigh fractures, which are permanently reduced and fixed by implanting different devices such as plates, nails, screws, wire, and needles [24].

A surgical intervention budget was used in a private clinic to calculate the direct cost of osteoporotic hip fracture in the private system.

To calculate the national direct cost of osteoporotic hip fracture, hospital discharges were multiplied by osteoporotic hip fractures in adults aged 45 years or more in 2017 by the individual cost of managing a hip fracture.

## 3. Results

**Figure 1:**
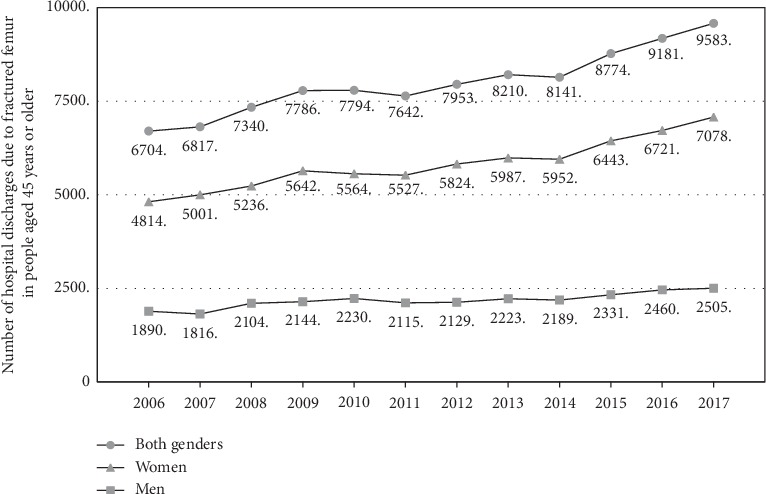
Trend of the hospital discharge due to fractures of the femur (code S72) in people aged 45 years or more by sex in Chile, 2016-2017.

Between 2006 and 2017, the number of hospital discharges due to osteoporotic hip fracture (S72) in adults aged 45 years or more has increased progressively, registering 9.583 hospital discharges for this cause in 2017, which corresponds to 50% more than those recorded in 2006. In Chile, most of the osteoporotic hip fractures occur in women, representing approximately 75% of these fractures in 2017. The progressive rise in hip fractures is observed in both men and women. In women, there was an increase from 4.814 in 2006 to 7.078 in 2017, and in men from 1.890 in 2006 to 2.505 during the same period (Figure 1).

**Figure 2:**
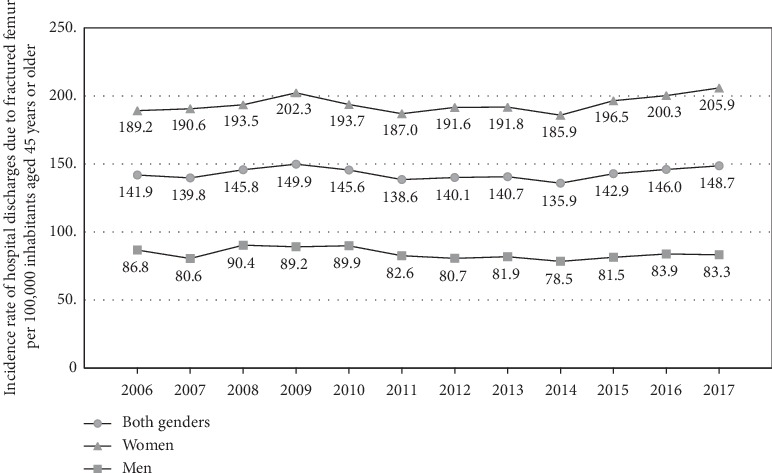
Incidence of the hospital discharge due to fractures of the femur (code S72) in people aged 45 years or more by sex in Chile, 2016-2017.

The incidence rate of hospital discharges due to osteoporotic hip fracture in people aged 45 years or more was 189.2 per 100.000 people in 2006 and 205.9 in 2017. In men in 2006, the rate was 86.8 per 100.000 and 83.3 per 100.000 in 2017; in women, the rate in 2006 was 141.9 per 100.000 and 148.7 per 100.000 in 2017 (Figure 2).

In 2017, 11,402 discharges were recorded due to fractures of the femur, in which 84% of these (9.583) occurred in adults aged 45 years or more and 73% occurred in adults aged 65 years or more (8.322). Osteoporotic hip fracture was considered as the sum of fractures of the femoral neck (S720), pertrochanteric fractures (S721), and subtrochanteric fractures (S722) in adults 45 years of age or older, totaling 7,934 osteoporotic hip fractures in 2017, which represented 94% of hospital discharges for this cause (Table 1).

In 2017, the individual cost of managing an osteoporotic hip fracture in the public system in Chile was USD$ 3.919. On the other hand, in the private health system, the cost was approximately USD$ 9.092 (Table 2).

The cost in the private health system is variable since it depends on the health program of each patient. The national average of days of stay for hip fracture was 15 days, and so the daily cost was calculated according to the day bed and multiplied by 15. The cost for the country adding up all the health systems was USD$ 33.721.230 (approximately, 34 million dollars) (Table 3).

## 4. Discussion

Worldwide, the population ages quickly; in 2015, the United Nations report on the aging of the world's population indicated that the fastest increase in the number of older people will occur in Latin America and the Caribbean, with the increase in the population of 60 years being estimated at 71% or greater by 2050 (from 71 million in 2015 to 200 million in 2050) [25]. By 2050, 12.5% of all hip fractures worldwide are expected to occur in this region [26], and in the case of Chile, the number of osteoporotic fractures and the health expenditure associated with them are expected to increase significantly given the expected increase in the number of adults aged 60 years or more by 2050 (from 2.8 million in 2017 to 6.9 million in 2050) [1, 2].

Osteoporosis is a silent disease, as one of its first clinical manifestations is fracture. The hip fracture is most commonly used to evaluate osteoporotic fractures since they are all hospitalized for treatment, being an excellent parameter for analysis between different regions of the world [27]. In 1990, it was estimated that there were 1.6 million hip fractures worldwide [28]. Due to the aging of the population, there is an exponential growth especially in Asia of the number of hip fractures, with an annual incidence for 2050 greater than 6 million hip fractures [29, 30].

The highest incidence of hip fracture is observed in Denmark (439 per 100,000) and one of the lowest in Ecuador (123 per 100,000), in people over 50 years old in Denmark and in people over 60 years old in Ecuador [31, 32]. In western countries, approximately 3 of 4 hip fractures occur in women. The countries with the highest incidence are those in Northern Europe (Norway, Sweden, Iceland, and Ireland), followed by those in Central Europe, (Denmark, Belgium, Germany, Switzerland, and Austria), those in Eastern Europe (Czech Republic, Slovakia, and Hungary), and those in the Middle East (Iran and Oman). Other countries with high incidence rates of hip fracture are Argentina and Taiwan [33].

The Latin American region has intermediate rates of hip fracture incidence, being lower than that of Scandinavian countries and higher than that of Asian countries. In people over 50 years, Brazil has an incidence of 141 per 100,000 people, Argentina of 264 per 100,000 people, and Colombia of 104 per 100,000 people [34]. In Chile, the incidence of hip fractures is 149 per 100,000 people over 45 years. We can observe that the amount of discharges due to femoral fractures in the population aged 45 years or more has increased annually from 2006 to 2017. Meanwhile, the annual rate of incidence of hip fractures in people aged 45 years or more was 189.2 per 100,000 inhabitants in 2006 and 205.9 per 100.000 inhabitants in 2017; which may be explained by the progressive aging of the population, which may allow the quantity to increase faster than the incidence of osteoporotic fractures in the country. It is estimated that by 2050, the population of 60 years or more will increase to 32%, compared to the 16.2% that this age group represents in 2017 [2], so the incidence rate is likely to be maintained for the year 2050, but that significantly increases the amount of osteoporotic hip fractures nationwide, which will mean a large increase in health spending for osteoporotic fractures.

The total cost of osteoporotic hip fractures was estimated at approximately 34 million dollars, and the individual cost for those treated in the public health system is USD$ 4.000 dollars and USD$ 9.000 dollars for those who are treated in the private health system. The cost of surgical management of osteoporotic hip fracture is significantly higher in the private health system in Chile since the surgical procedure is performed in private clinics whose costs are very high compared to the public sector. There is a notable difference in the availability of medical coverage for the hip fracture between the public health system and the private health system since only 11% of the Chilean population is in the private health system.

Osteoporotic hip fractures can be considered from the age of 50, but since the classification of the DEIS included the people aged between 45 and 64 years, we had to include the people aged between 45 and 50 years (age group that may not be related to osteoporosis fractures), so we could include the people aged between 50 and 64 years (age group that may be related to osteoporosis fractures).

According to a report from the European Union, the cost of osteoporosis fractures would be approximately 25,000 million euros, figures that could double in the next 50 years due to the aging of the population. The direct cost of osteoporotic hip fracture is 1,256 euros in Poland, 9,996 euros in France, and 29,910 euros in Switzerland [35]. In Spain, the direct costs during the first year of an osteoporotic hip fracture are approximately 12,300 euros in Madrid and 6,500 in Andalucía [36]. The economic cost of the hip fracture is similar to that of an acute coronary syndrome ($ 32,345) and that of a stroke ($ 34,772) [37, 38].

Compared to South American countries, Chile has a health expenditure similar to the countries in the area. The expense for osteoporosis and osteoporotic fractures in Brazil is USD 92 million (2.8 times higher than in Chile) [17], for a population of 209 million (11.7 times higher than the Chilean); hospitalization expense for hip fracture and vertebrae in Argentina is USD 190 million (5.7 times higher than in Chile) [15], for a population of 44 million (2.5 times higher than the Chilean); hospital expenditure for treating hip fractures in Colombia is USD 51 million (1.5 times higher than in Chile) [19, 39], for a population of 49 million (2.8 times higher than the Chilean); fragility fracture spending in Mexico is USD 256 million (7.8 times higher than in Chile) [16], for a population of 130 million (7.3 times higher than the Chilean).

The annual expenditure associated with osteoporotic fractures and osteoporosis is estimated to be greater than that identified in this study (USD 34 million), as the expenditure identified in this study only quantifies the expenditure associated with the management of the osteoporotic hip fracture and not the expenditure on the annual monitoring of fracture, in other fracture types, in tests used for the diagnosis of osteoporosis, or in the treatment of osteoporosis. The costs of medical treatment during the year following the surgery in the US vary in a range from $21,559 to $44,200, which is influenced by the local health policy that influences the effectiveness of the handling of this pathology [10].

Within the limitations of the work, it can be mentioned that the complications of prolonged rest or surgical procedures and infection of the operative pathway, venous thrombosis, or pulmonary thromboembolism among others were not considered in the evaluation of the direct economic impact of hip fractures. Another limitation is including the population aged 45–50 years because they are not related to fragility fractures. The study does not specify whether it is the first or second fracture, and there may be a duplication of cases: almost half of the patients over 50 years admitted with a hip fracture previously suffered a fragility fracture [40]. The design of this study does not allow to identify the mechanism that triggers the fracture, nor the presence of a previous fracture. The number of cases could be dismissed if the patients had been treated outside the hospital [41].

## 5. Conclusions

The osteoporotic hip fracture represents a significant economic impact not only on a personal level but for the entire Chilean health system, with a marked difference between public and private health systems.

In women over 50 years old, the group that suffers the majority of osteoporotic fractures, Chile has an incidence of hip fractures per 100,000 people similar to that of Spain, Mexico, Estonia, Poland, Brazil, and Thailand (incidence close to 200 cases for every 100,000 women over 50 years old); this corresponds to the group of moderate incidence worldwide (200–300 cases per 100,000) [41].

The cost of the initial hospitalization in the treatment of osteoporotic hip fractures in the public system of Chile is similar to that of Latin American countries such as Uruguay and Argentina (3-4 thousand dollars) [19], while the cost in the private health system is similar to that of European countries such as Belgium (9 thousand dollars) [27], being very close to the world average (10 thousand dollars) [10].

## Figures and Tables

**Table 1:** Hospital discharges of the year 2017 according to cause and age.

	All ages	45 years and older
Femoral fracture (S72)	11.402	9.583
Femoral neck fracture (S720)	6.992	6.577
Pertrochanteric fracture (S721)	1.197	1.154
Subtrochanteric fracture (S722)	254	203
Hip fractures S720 + S721 + S722	8.443	7.934

**Table 2:** Cost of osteoporotic hip fracture in the private system.

	Quantity	Unit cost (CLP)	Total cost (CLP)	Total cost (USD)
Clinical supplies (from preoperative to postoperative)	1	2.300.000	2.300.000	3.270
Medications per day of hospitalization	15	79.000	1.185.000	1.685
Anesthesia	1	155.000	155.000	220
Medical fees (first surgeon, second surgeon, and anesthetist)	1	808.000	808.000	1.150
Single bed day	15	95.000	1.425.000	2.027
Hospital pavilion rights	1	520.000	520.000	740
Individual cost of osteosynthesis of the thigh	1	6.393.000	6.393.000	9.092

**Table 3:** Cost of osteoporotic hip fracture nationwide.

	Public system	Private system	Other types of health system	Total
Percentage of osteoporotic hip fracture discharges according to health system	86.7%	6.4%	6.9%	100%
Hospital discharges due to osteoporotic hip fracture according to health system	6.879	508	547	7.934
Individual cost of osteoporotic hip fracture according to health system (CLP)	2.755.060	6.393.000	2.755.060	
Individual cost of osteoporotic hip fracture according to health system (USD)	3919	9092	3919	
National cost of osteoporotic hip fracture according to health system (CLP)	18.952.057.740	3.247.644.000	1.507.017.820	23.706.719.560
National cost of osteoporotic hip fracture according to health system (USD)	26.958.801	4.618.736	2.143.693	33.721.230

## Data Availability

All data are stored as electronic databases in the Department of Health Statistics and Information (DEIS) of the Ministry of Health of Chile and can be accessed through their website.
